# Identification and characterization of circRNAs in the skin during wool follicle development in Aohan fine wool sheep

**DOI:** 10.1186/s12864-020-6599-8

**Published:** 2020-02-28

**Authors:** Ranran Zhao, Nan Liu, Fuhui Han, Hegang Li, Jifeng Liu, Lanlan Li, Guoyi Wang, Jianning He

**Affiliations:** 1 College of Animal Science and Technology, Qingdao Agricultural University, Qingdao, 266109 China; 2 Aohan Fine Wool Sheep Stud Farm, Chifeng, 024000 China

**Keywords:** Aohan fine wool sheep (AFWS), Skin, Wool follicle, Pathways, Circular RNA

## Abstract

**Background:**

Aohan fine wool sheep (AFWS) is a historically bred fine wool sheep, cultivated in China. The wool has excellent quality and good textile performance. Investigating the molecular mechanisms that regulate wool growth is important to improve wool quality and yield. Circular RNAs (circRNAs) are widely expressed non-coding RNAs that can act as competitive endogenous RNAs (ceRNAs) to bind to miRNAs. Although circRNAs have been studied in many fields, research on their activity in sheep wool follicles is limited. To understand the regulation of circRNAs in the growth of fine wool in sheep, we used RNA-Seq to identify circRNAs in sheep shoulder skin samples at three developmental stages: embryonic day 90 (E90d), embryonic day 120 (E120d), and at birth (Birth).

**Results:**

We identified 8753 circRNAs and found that 918 were differentially-expressed. We then analyzed the classification and characteristic of the circRNAs in sheep shoulder skin. Using Gene Ontology (GO) and Kyoto Encyclopedia of Genes and Genomes (KEGG), we identified the source genes of circRNAs, which were mainly enriched in cellular component organization, regulation of primary metabolic processes, tight junctions, and the cGMP-PKG and AMPK signaling pathways. In addition, we predicted interactions between 17 circRNAs and eight miRNAs, using miRanda software. Based on the significant pathways, we speculate that circ_0005720, circ_0001754, circ_0008036, circ_0004032, circ_0005174, circ_0005519, and circ_0007826 might play an important role in regulating wool follicle growth in AFWS. Seven circRNAs were randomly selected to validate the RNA-Seq results, using qRT-PCR.

**Conclusion:**

Our results provide more information about circRNAs regulation of wool follicle development in AFWS, and establish a solid foundation for future research.

## Background

Wool is a source of high-quality textile raw materials derived from animals, that has a significant impact on the national economy. Improving the production of high-quality fine wool has become a hot topic in recent years. Wool growth is a very complex physiological and biochemical process, influenced by genetics, the environment, and nutrition. Wool grows from hair follicles (HF), and its yield and quality are closely related to the development of wool follicles. These are complex organs of the skin that are capable of self-regeneration, and their structure plays a very important role in their periodic growth process. Mammalian hair follicles are divided into primary hair follicles (PF) and secondary hair follicles (SF). It is the SF that is producing fine wool. Wool follicle morphogenesis involves the coordination of a series of signaling pathways that connect epidermis and dermis. The development of hair follicles is regulated by various signaling pathways, such as Wnt, sonic hedgehog (SHH), notch, bone morphogenic protein (BMP), and fibroblast growth factor (FGF). Various downstream signaling molecules, such as β-catenin, Msx1, and Msx2, are involved in hair follicle morphogenesis [1]. In recent years, many studies have indicated that non-coding RNAs act as important post-transcriptional regulators of gene expression during hair follicle development, including microRNAs (miRNAs), circular RNAs (circRNAs), and long non-coding RNAs (lncRNAs). LncRNA acts on the Wnt signaling pathway and affects hair follicle growth and development [2]. Non-coding RNA has also been shown to regulate wool fineness and growth of SF in cashmere goats [3].

CircRNAs are a novel type of noncoding RNA that regulate transcriptional and post transcriptional genes expression [4, 5]. They are typically generated by back-splicing from exons of protein-coding genes and their 5′ and 3′ ends join together to form a ring. Because of the absence of 5′ and 3′ open ends, they are more stable than linear RNAs and are resistant to RNase R digestion [6, 7]. CircRNAs are widely distributed in mammalian cells and endogenously regulate genes expression [8]. They have specificity for tissue, developmental stage, and cell type [9, 10]. CircRNAs act as miRNA molecule sponge [11], regulate gene transcription [12, 13], interact with RNA-binding proteins [14, 15], and translate proteins [16].

Recently, studies have found that exposure to melatonin disturbs a key secretion signal in goat hair follicle stem cells, and consequentially disturbs normal goat hair follicle development [17]. CircRNA has been shown to participate and regulate human skin tissue regeneration [18]. It was also shown that it has tissue-specific and stage-specific expression in chicken follicle granulosa cells. As such, circRNAs are useful when investigating the regulatory mechanisms of follicular growth [19]. Research on the hair follicle cycle in the Angora rabbit revealed the existence of a lncRNA/circRNA-miRNA/mRNA network and has shown that non-coding RNAs (ncRNAs) play an important role in regulating the HF cycle [20]. In another recent study, a total of 12,468 circRNAs and 9231 differentially-expressed circRNAs were identified in the estrus and anestrus states of the sheep pituitary system [21]. However, there are few reports on the involvement of circRNA in the development of sheep wool follicles.

Aohan fine wool sheep (AFWS) is a sheep breed in China that produces excellent wool quality, with good textile process performance. Increased understanding of the function of genes involved in wool follicle development could assist in selective breeding for specific traits and thus improve wool yield and quality [22]. In a previous study on wool follicle development in AFWS, we showed that a small number of SF could already be observed at embryonic day 90 (E90d), and a large number of SF were found at embryonic day 120 (E120d). Secondary wool follicles had mostly completed development by the time of birth (Birth) [23].

To date we have only a very limited understanding of circRNA expression in AFWS follicles. To study the relationship between circRNA and changes in wool follicle at different developmental stages in sheep, RNA-Seq was used to detect the expression profiles of circRNA in skin tissue from AFWS at E90d, E120d, and Birth. Our results indicate that circRNA plays an important role in the formation of sheep wool follicles.

## Results

### Secondary wool follicle growth process

**Fig. 1:**
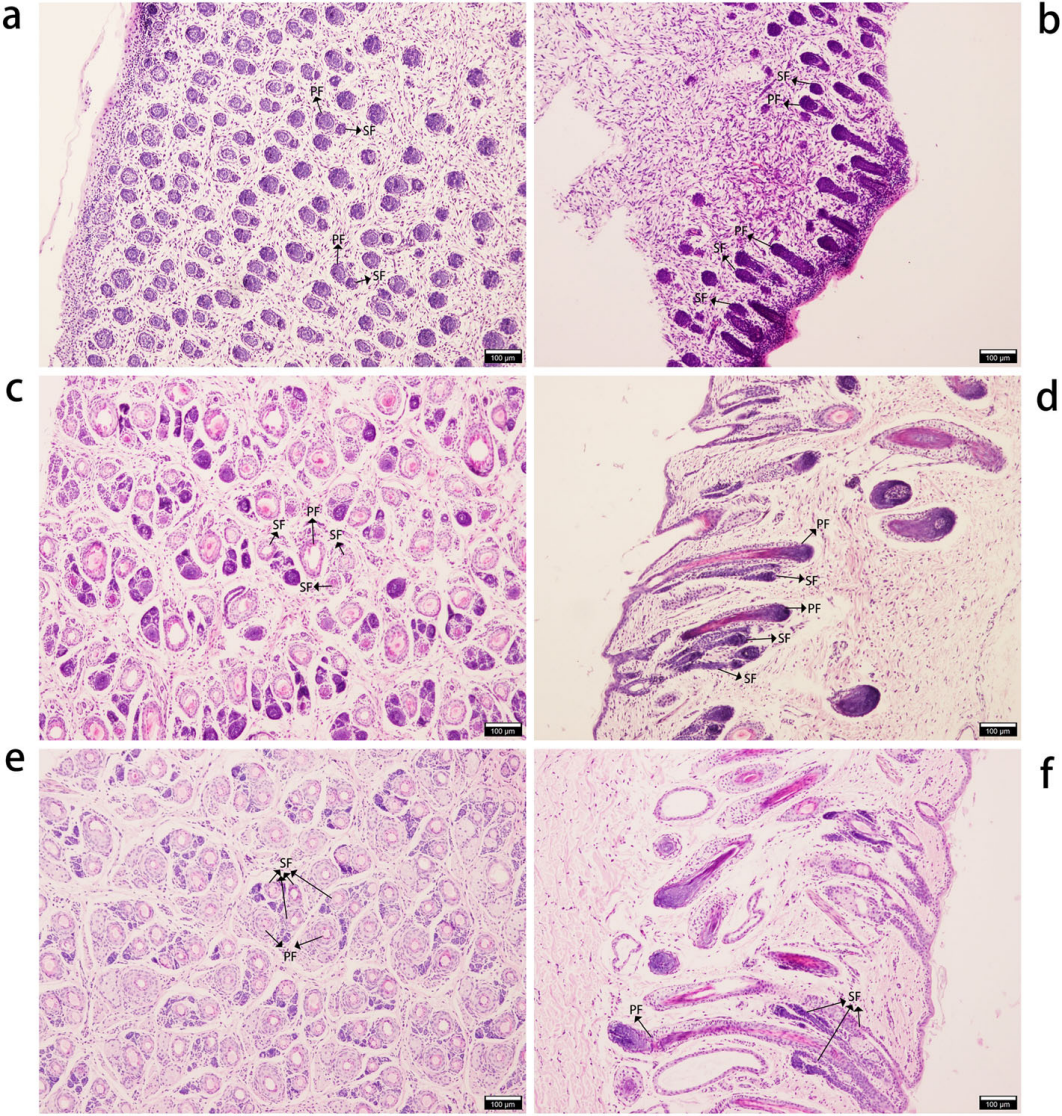
Hematoxylin-Eosin staining of sheep wool follicles at different developmental stages. Tissue morphology of secondary wool follicles at different stages was determined. Horizontal and longitudinal slices of tissue at E90d (**a**, **b**), E120d (**c**, **d**), and Birth (**e**, **f**) stages. PF: Primary wool follicle; SF: Secondary wool follicle

Hematoxylin and eosin (H&E) staining at E90d showed primary- and early secondary-stage wool follicles (Fig. 1a). From observing wool follicles at this stage, it is clear that PFs occur early, the bulbs are large, the wool follicles are long and have accessory structures such as sweat glands, sebaceous glands, and the arrector pili muscles. Secondary wool follicles at this stage are smaller and grow nearer to the epidermis than the PFs (Fig. 1b). At E120d, the SFs are separated from PFs and arranged in parallel to them (Fig. 1c, d). By birth, some of the SFs have matured and their wool has passed through the body surface (Fig. 1e, f).

### Sequencing and mapping of the sheep skin tissue transcriptome

**Fig. 2:**
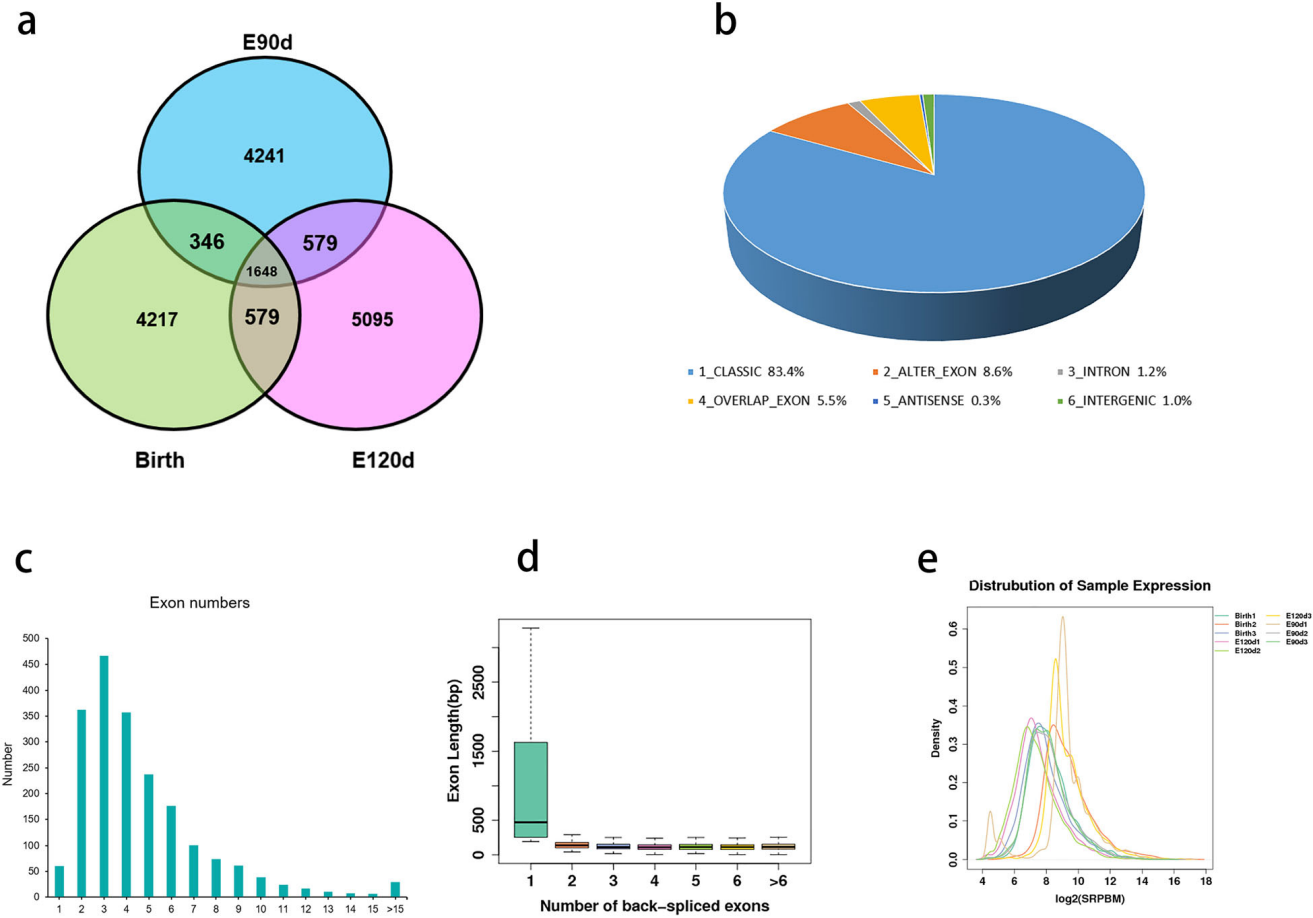
General characteristics of circRNAs in Aohan fine wool sheep skin. **a** Venn diagram showing circRNA annotated in sheep shoulder skin during the three developmental stages. **b** Classification of 4123 circRNAs screened in this study. Expression pattern of circRNAs at the three developmental stages. Exon number (**c**) and length (**d**), and expression density (**e**) of the samples

To examine the circRNAs expression profiles in sheep skin at different developmental stages, we performed RNA Integrity Number (RIN) tests on nine sheep skin tissue samples, three from each of the three developmental periods (E90d, E120d, and Birth). The RIN values ​​of the samples are listed in Additional file 1 (Table S1). Results show that the RNA quality met the minimum requirements for sequencing. Library was thus constructed and the samples were sequenced. Raw reads were acquired via Illumina sequencing, which were then processed to remove rRNA, low-quality sequences, and junction contamination, among other processing. All subsequent analyses were based on these processed clean reads. These reads were mapped to the sheep genome. The overall assessment of sequencing data is listed in Additional file 1 (Table S1). A total of 8753 candidate circRNAs and 3119 source genes were identified (Additional file 2: Table S2), 1648 of which (18.8%) were expressed at all developmental stages (Fig. 2a). The 30 highest-expressed circRNAs in each group are listed in Table 1. Based on their location in the genome, the 8753 circRNAs were classified into six types: (1) Classic: when the formation site of the circRNA was exactly on the boundaries of exons (83.4%); (2) Alter-exon: when one end of the circRNA formation site was on the exon boundary, and the other end was inside the exon (8.6%); (3) Intron: when the formation site of the circRNA was completely in the intron region (1.2%); (4) Overlap-exon: when the formation site of the circRNA spanned the exon region (5.5%); (5) Antisense: when the circRNA was formed by the antisense strand of the gene (0.3%); (6) Intergenic: when the formation site of circRNA was completely inside the intergenic region (1.0%) (Fig. 2b). circRNAs typically comprised of two to four exons (Fig. 2c). In circRNAs with only one exon, the length of the exon was found to be significantly longer than that of a circRNAs comprised of multiple exons (Fig. 2d). The peak gene density, based on the expression of circRNAs in all samples, was between 0.3 and 0.4 (Fig. 2e).

**Table 1:**
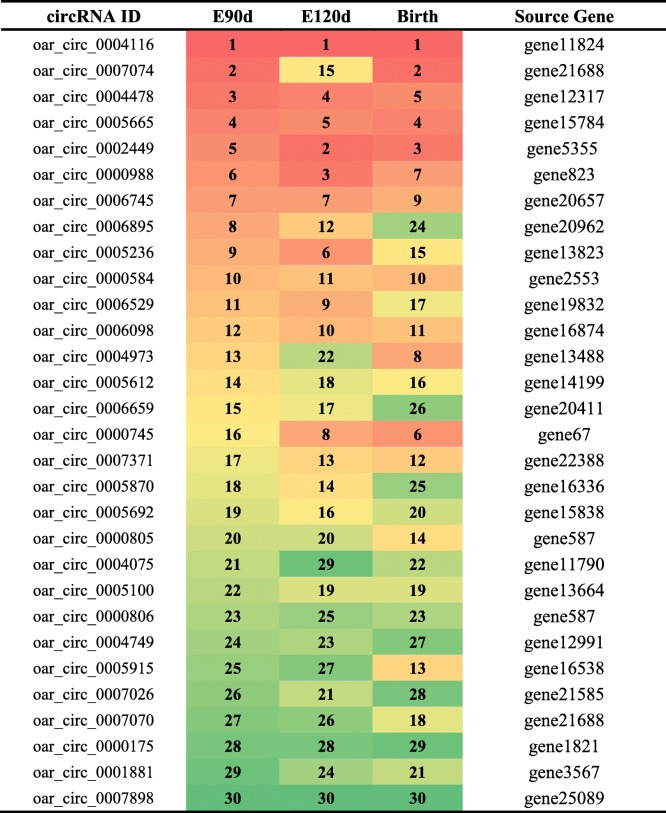
The top 30 expressed circRNAs during the three developmental stages of wool follicles

The 30 highest-expressed circRNAs in each group. Red color indicates a higher expression level of circRNAs and green color indicates lower expression level. The numbers 1 to 30 in each column represent the expression level of circRNA in descending order

*oar Ovis aries* reference

### Identification of differentially-expressed circRNAs

**Fig. 3:**
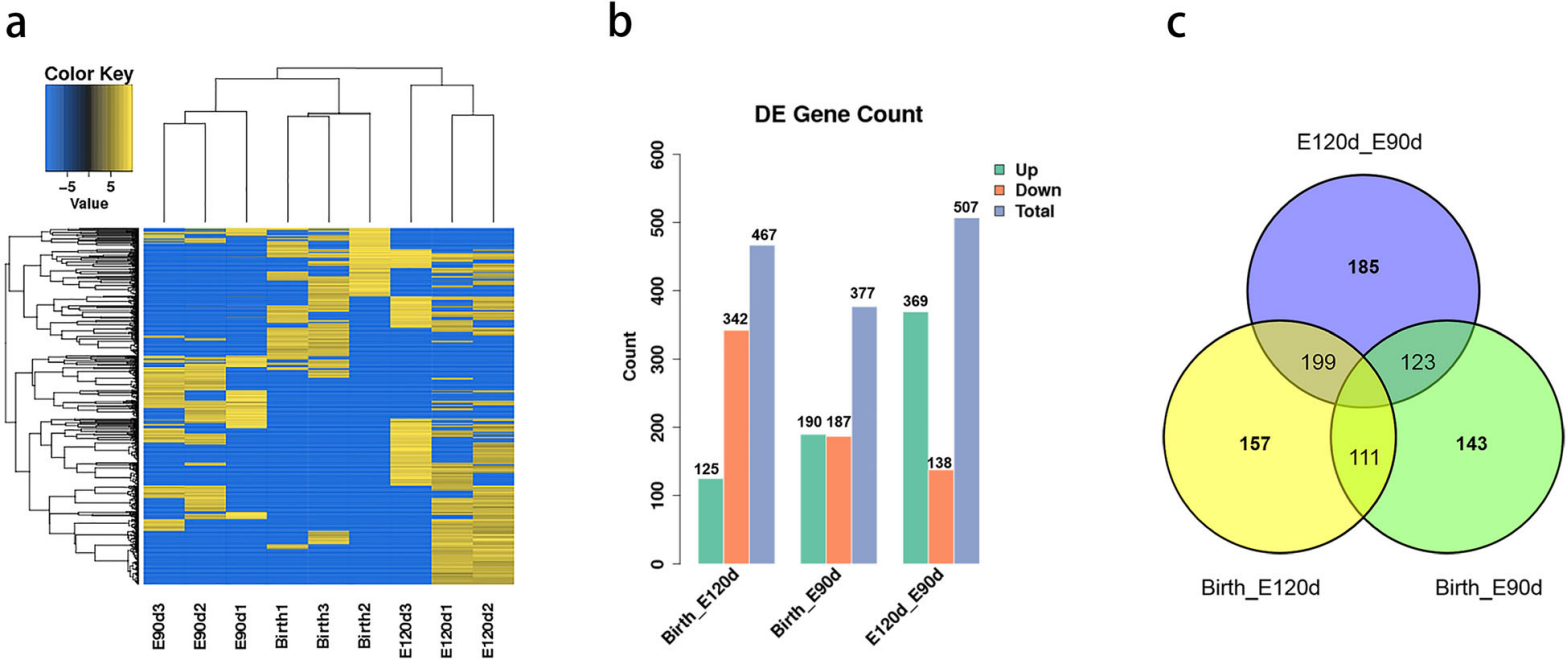
Identification of differentially-expressed circRNAs. **a** Heatmap of differentially-expressed circRNA. Yellow indicates that the circRNA had a higher expression level, and blue indicates that the circRNA had a lower expression. **b** Differentially-expressed circRNA statistics, the number of up and down-regulated circRNAs in each group has been marked on the graph. **c** Differentially-expressed circRNAs in pairwise comparisons groups

Based on the criterion of differentially-expressed circRNAs, clustering maps (Fig. 3a) were used to illustrate their distribution. Significantly differentially-expressed circRNAs in the figure are in yellow (upregulated expression) or blue (downregulated expression). The number of differentially-expressed circRNAs in the three developmental stages are displayed in Fig. 3b, c. We detected 377 differentially-expressed circRNAs and 314 source genes by comparing Birth and E90d, 467 differentially-expressed circRNAs and 383 source genes by comparing Birth and E120d, and 507 differentially-expressed circRNAs and 417 source genes by comparing E120d and E90d (Additional file 3: Tables S3A, S3B, S3C).

Among the DEGs (Differentially expressed genes), circ_0004932 and circ_0004936 were mapped to gene 13,410 (*TRPS1*). It has been reported that *Trps1* is involved in the growth and development of hair follicle cells [24]. Similar to circ_0004932 and circ_0004936, other circRNAs were also associated with hair follicle growth. These included circ_0000997 and cir_0000999 that were mapped to source gene 851 (*VAV3*), and circ_0001520 and circ_0001524 that were mapped to source gene 3008 (*TMEFF1*) [25, 26]. We also found that the expression level of circ_0006736 at E120d and Birth stages was significantly higher than E90d. It might therefore play a role in the growth, development, and maturation of SF. Mapping results showed that gene 20,646 (*SMAD1*) is the source gene of circ_0006736. This gene can control the transformation of early hair follicle morphology by controlling the activity of stem cells [27]. The expression levels of circ_0005454 and circ_0005453 at E120d were significantly higher than E90d. We have also noted that SFs grew significantly during the period between E90d and E120d. Based on these observations, we speculate that circ_0005454 and circ_0005453 participate in the growth of SF. Expression of circ_0004116 was high at all three developmental stages. It therefore might be active through the entire wool follicle growth process, including that of both PF and SF. In the future, we hope to further study the function of *RFX7*, the source gene of circ_0004116, in AFWS wool follicles development.

### Gene ontology and Kyoto encyclopedia of genes and genomes pathway enrichment analyses

**Fig. 4:**
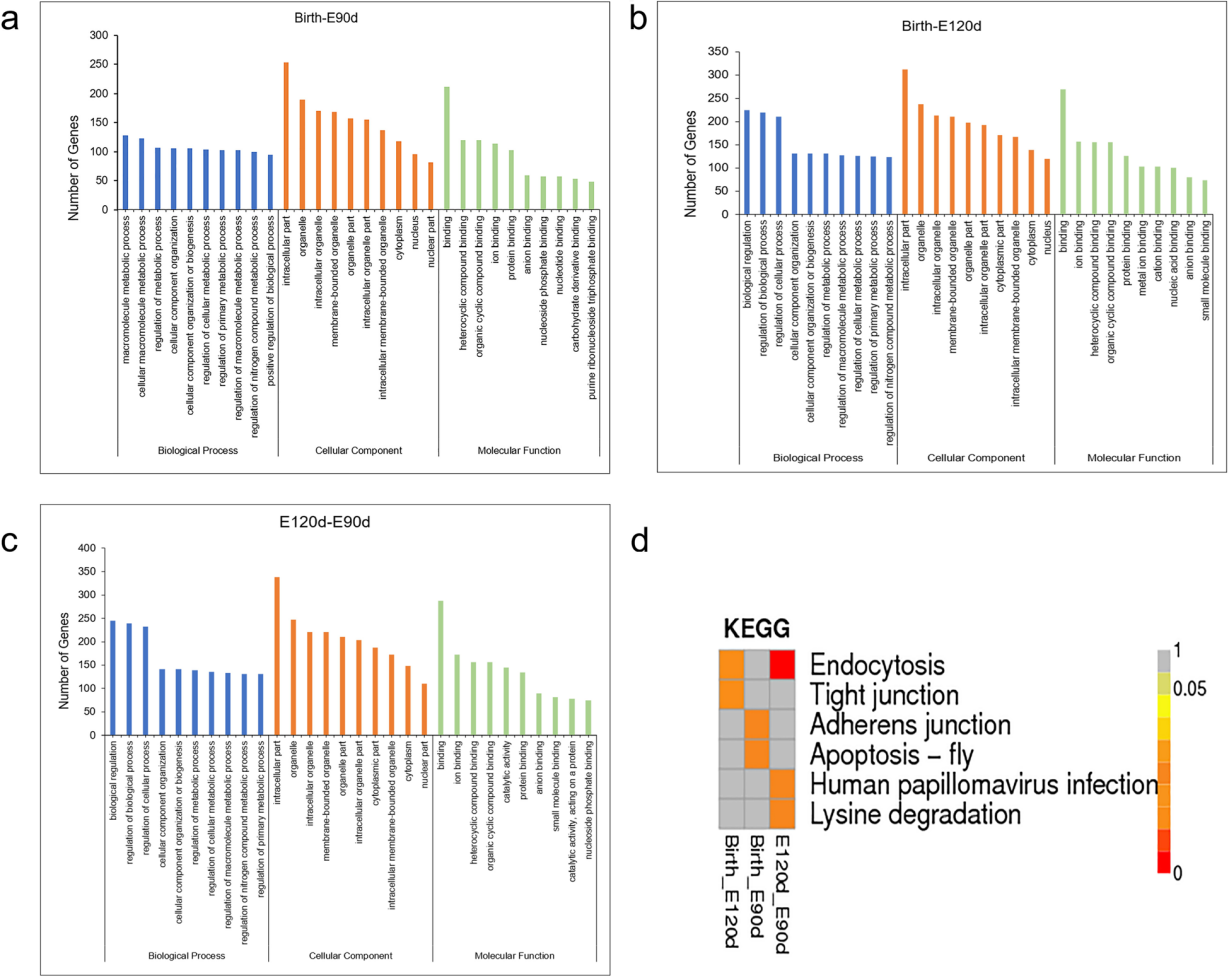
Function analysis of source genes of differentially-expressed circRNAs. **a** Gene Ontology analysis of circRNA host genes between Birth and E90d. **b** Gene Ontology analysis of circRNA host genes between Birth and E120d. **c** Gene Ontology analysis of circRNA host genes between E120d and E90d. **d** The Kyoto Encyclopedia of Genes and Genomes heat map of differentially-expressed circRNAs

The function of circRNA is reflected through their source gene. It thus can be further studied by analyzing the Gene Ontology (GO) terms of their source genes. Based on statistical analysis of differentially-expressed circRNAs and their source genes (Additional file 3: Table S3), the top ten terms of candidate genes in each comparison group were selected for mapping (Fig. 4a-c). Detailed information is listed in Additional file 4 (Tables S4A, S4B, S4C). The most significantly enriched GO terms were: cellular component organization (GO: 0016043), regulation of primary metabolic process (GO: 0080090), intracellular part (GO: 0044424), intracellular organelle (GO: 0043229), membrane-bounded organelle (GO:0043227), and protein binding (GO: 0005515).

**Fig. 5:**
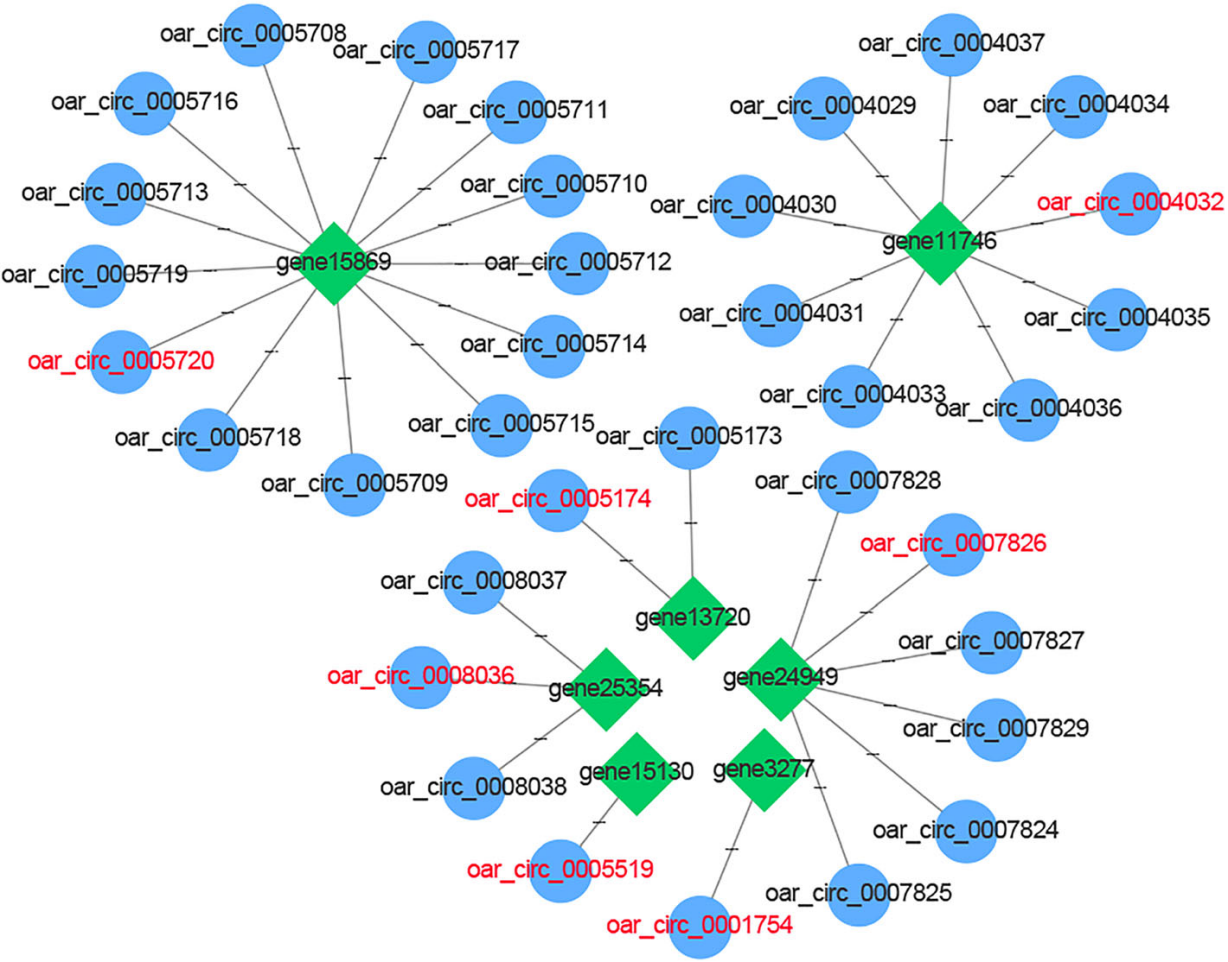
Interactions between circRNAs and source genes. Green node represents source gene, blue node represents circRNA, and circRNAs highlighted in red were derived from our candidate genes. Edge denotes the relationship between circRNA and source gene

To predict the pathways of the significantly enriched source genes, we performed an enrichment analysis using the Kyoto Encyclopedia of Genes and Genomes (KEGG) pathway analysis (Fig. 4d, Additional file 5: Table S5A, S5B, S5C). Six significantly enriched pathways were identified. These were endocytosis, lysine degradation, apoptosis, human papillomavirus infection, adherence junction, and tight junction. The six pathways involve 55 enriched source genes and their corresponding 255 circRNAs (Additional file 6: Table S6A). Among the 55 source genes, seven are associated with wool follicle growth. There were 35 circRNAs associated with these seven source genes (Additional file 6: Table S6B). Of these, seven were found to be significantly differentially-expressed in our study: circ_0005720 from source gene 15,869 (*AKT3*), circ_0001754 from source gene 3277 (*TGFBR1*), circ_0008036 from source gene 25,354 (*SMAD2*), circ_0004032 from source gene 11,746 (*SOS2*), circ_0005174 from source gene 13,720 (*RB1*), circ_0005519 from source gene 15,130 (*EZH1*), and circ_0007826 from source gene 24,949 (*FGFR2*). A network describing the connections between the source genes and circRNAs was constructed (Fig. 5).

### Target miRNAs of differentially expressed circRNAs at the different developmental stages in sheep

**Fig. 6:**
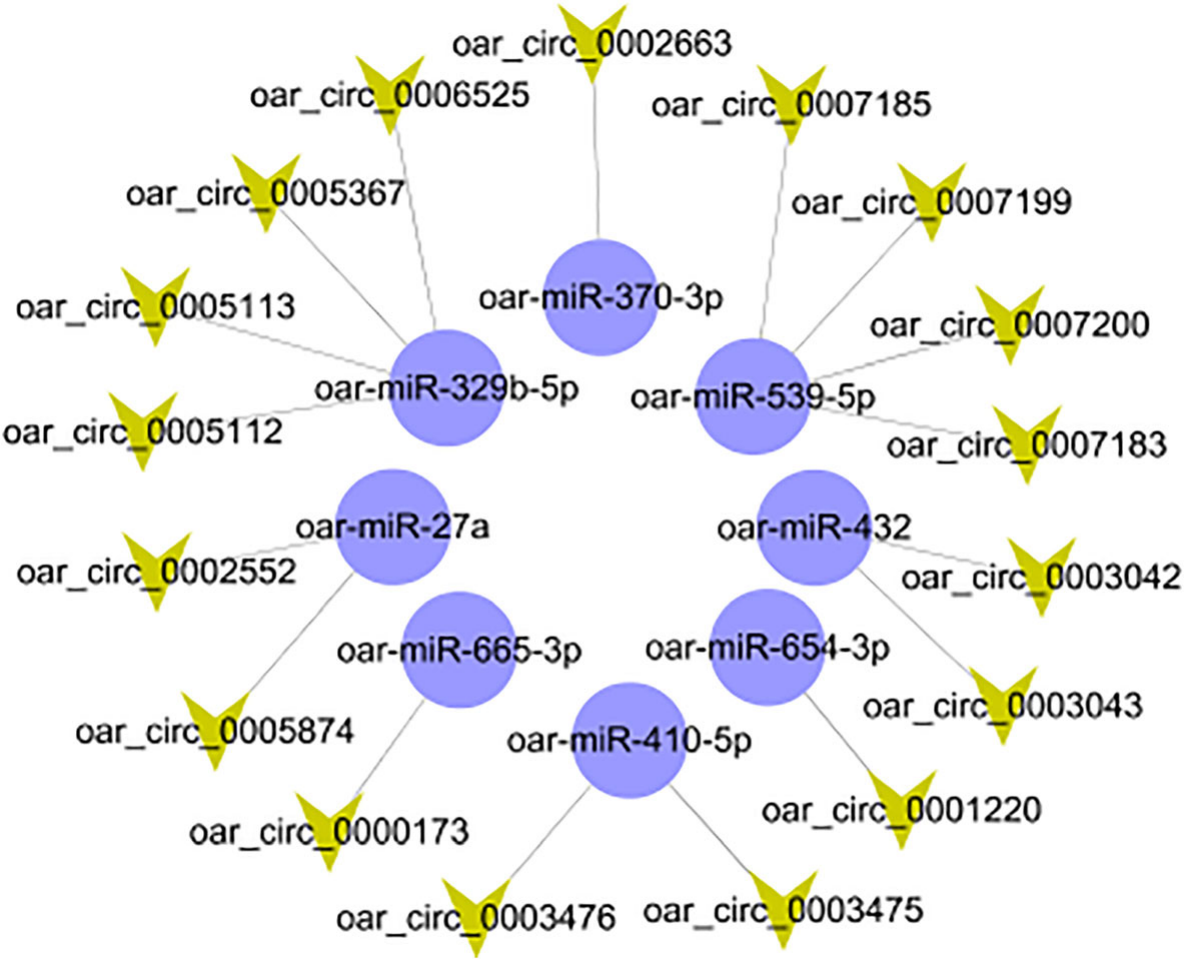
Interactions between circRNAs and miRNAs. Yellow node represents circRNA, purple node represents miRNA. Edge denotes the relationship between circRNA and miRNA

To further understand the functions of circRNAs, the miRanda software was used to predict the interactions between the identified circRNAs and miRNAs. A total of 17 circRNAs and eight miRNAs were identified, and the relationships between them were constructed into a network (Fig. 6, Table 2). For example, circ_0003042 is significantly differentially-expressed between Birth and E120d. This circRNA was predicted to interact with miR-432. By binding all available miR-432, circ_0003042 prevents miR-432 from exerting its function, effectively acting as “miRNA sponge.”

**Table 2:** Prediction of targeting relationship between circRNA and miRNA

circRNA ID	miRNA Name
oar-circ_0002552	oar-miR-27a
oar-circ_0005874	oar-miR-27a
oar-circ_0005112	oar-432-5p
oar-circ_0005113	oar-miR-329b-5p
oar-circ_0005367	oar-miR-329b-5p
oar-circ_0006525	oar-miR-329b-5p
oar-circ_0002663	oar-miR-370-3p
oar-circ_0003475	oar-miR-410-5p
oar-circ_0003476	oar-miR-410-5p
oar-circ_0003042	oar-miR-432
oar-circ_0003043	oar-miR-432
oar-circ_0007183	oar-miR-539-5p
oar-circ_0007185	oar-miR-539-5p
oar-circ_0007199	oar-miR-539-5p
oar-circ_0007200	oar-miR-539-5p
oar-circ_0001220	oar-miR-654-3p
oar-circ_0000173	oar-miR-665-3p

*oar Ovis aries* reference

### Validation of circRNAs expression by qRT-PCR

**Fig. 7:**
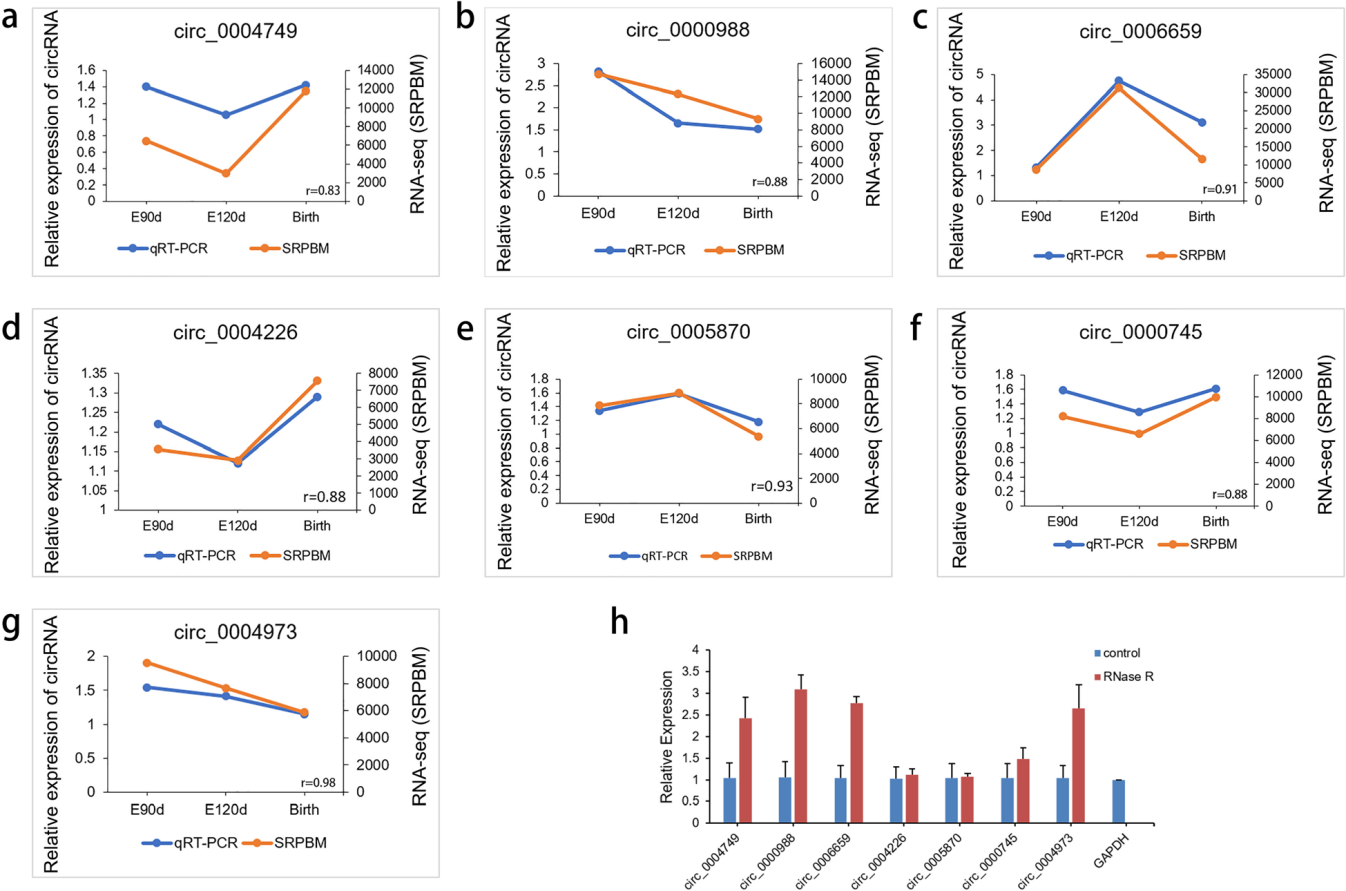
Validation and RNase R resistance of circRNAs. **a**-**g** Experimental and sequencing validation of seven circRNAs. Correlation between RNA-Seq and qRT-PCR is represented by r. **h** After RNase R treatment, the expression level of circRNAs and GAPDH were measured. The X-axis indicates circRNAs, and the Y-axis indicates the relative expression level of circRNAs and GAPDH. Error bars indicates ±SD

To validate the expression levels of differentially-expressed circRNAs, we randomly selected seven highly expressed circRNAs and detected their expression levels by qRT-PCR (Additional file 7: Table S7). These results were consistent with the trends observed in the RNA-Seq data. The correlation results for all circRNAs were r > 0.8, indicating that the RNA-Seq is reliable (Fig. 7a-g). As can be seen in Fig. 7h, the circRNAs we selected could resist RNase R digestion, while the linear RNA in the sample (GAPDH) could not. After RNase R digestion, expression of the seven circRNAs did not significantly decrease. On the contrary, most of them actually increased. We speculated that circRNAs were relatively enriched, and the efficiency during reverse transcription has relatively improved. The relative expression levels quantified by qRT-PCR have therefore also increased. RNase R digestion basically increased the purity of circRNAs. The results show that circRNAs can resist the digestion of RNase R, while linear RNAs cannot.

## Discussion

In this study, we investigated the expression of circRNAs in sheep skin wool follicles at different developmental stages. Using RNA-Seq technology, we obtained 8753 circRNAs at the three developmental stages in sheep. Of the identified differentially-expressed circRNA and source genes, respectively, 377 and 314 were detected by comparing Birth and E90d, 467 and 383 were detected by comparing Birth and E120d, and 507 and 417 were detected by comparing E120d and E90d. In a study on the three hair follicle cycle stages of Angora rabbit, performed using RNA sequencing, 247 differentially-expressed circRNAs (128 upregulated and 119 downregulated) were found. It was suggested that several circRNAs, including novel ones such as circ_0004876, circ_0005177, and circ_0026326, might play a role during hair follicle cycle [20]. Many mammalian species have similar hair follicle growth patterns, and a number of them have been studied, including goat [28], rat [29], and human [30]. The main purpose of analyzing sheep wool follicle circRNAs was to reveal factors that might play a role in wool growth, thereby elucidating the underlying molecular mechanisms.

To further investigate potential circRNAs mechanisms of action, we applied GO and KEGG analyses. In GO annotation, the number of DEGs between any two stages exhibiting significant differences, reflecting a cumulative effect on phase traits. It was found that source genes of the differentially-expressed circRNAs function primarily in biological processes. These included terms under Cellular Component: cellular component organization (GO: 0016043), regulation of cellular processes (GO: 0050794), cellular macromolecule metabolic processes (GO: 0044260), intracellular organelle (GO: 0043229) and organelle part (GO: 0044422); and Molecular Function: binding (GO: 0005488), ion binding (GO: 0043167), and heterocyclic compound binding (GO: 1901363). These findings indicate that the different source genes of circRNAs at the three developmental stages play a significant role in of wool follicle cells’ formation, playing functions related to GO terms such as regulatory of metabolic processes. Some hair follicle-related GO terms were also enriched in our study, including regulation of hair cycle (GO: 0042634), skin development (GO: 0043588), hair follicle development (GO: 0001942), regulation of epidermis development (GO: 0045682) and hair cycle process (GO: 0022405). Some of them were reported to participate in the growth of hair follicle, and might be important research targets [20]. It was found that circRNAs expression profiles usually follow those of their source gene [31, 32]. Our study suggests that the identified circRNAs might be associated with these GO terms, however further validation is required.

The KEGG is a pathway database for systematic analysis of gene function. The results we obtained suggest that multiple signaling pathways form a complex regulatory network during wool follicle development. It was reported that human papillomavirus infection [33], adherence junction [34], and tight junction [35] signaling pathways participate in the growth and development of hair follicles. In our study, seven circRNAs (circ_0005720, circ_0001754, circ_0008036, circ_0004032, circ_0005174, circ_0005519, and circ_0007826) were identified based on the significant KEGG pathways. The source genes of these circRNAs (*AKT3*, *TGFBR1*, *SMAD2*, *SOS2*, *RB1*, *EZH1*, and *FGFR2*) were reported to participate in the growth process of hair follicles [36–43]. Comparison between our results and those of previous studies suggests that the selected circRNAs might play an important role in the signaling pathways during different stages of SF development in AFWS. However, further research is required to identify the exact related mechanisms.

Although some signaling pathways, such as the Hedgehog [44], MAPK [45], FoxO [46], TGF-β [47], NF-κB [48], TNF [49], and Wnt [50] were found not to be significant in our study, the source genes of these were previously reported to regulate the development of skin and hair [51]. Wnt, Hedgehog and NF-κB/Edar pathways were found to be indispensable in the process of hair follicle growth [52]. Edar signaling pathway is involved in controlling the development and circulation of HFs. The interaction between ectodysplasin receptor (EDAR) and bone morphogenetic protein (BMP) signaling and transcription is at the core of the PF model [53, 54]. Studies have shown that Wnt/β-catenin signaling is important for NF-κB activation, and that Edar can directly target Wnt. The Wnt/β-catenin and EDA/Edar/NF-κB signaling pathways play an important role in the initiation and maintenance of PF placodes [55]. Research on the relationship between these signaling pathways is still incomplete, and what is known about the molecular mechanisms involved in HF development has been derived primarily from studies conducted in mice and humans [56, 57].

Recent studies have found that some circRNAs have multiple binding sites for miRNAs (such as CDR1as and miR-7, SRY and miR-138) [9, 58]. As circRNAs are unable to directly regulate their target genes, they function as “miRNA sponge.” It has been reported that circRNAs participate in many biological processes by acting as miRNA sponges, thereby removing the inhibitory effects of miRNAs on their target genes [58]. In recent years, miRNAs have been studied from a variety of aspects related to HF growth and cell cycle [59, 60]. A study has shown that circRNAs can regulate gene expression through a circRNA-miRNA-mRNA pathway [31]. Another pioneering study has demonstrated that miRNAs are differentially-expressed between fat-tailed and short-tailed sheep breeds [61]. However, there remains lack of research on circRNA in sheep SF at different developmental stages. A circRNA-miRNA network, which contains 17 circRNAs and eight miRNAs, was constructed based on the results of the KEGG pathway analysis. This network can help us better understand the characteristics of sheep SF at the different developmental stages. In a study on proliferation of thyroid carcinoma, miR-370-3p was reported to act as a target of circRNA_NEK6 via the Wnt signaling pathway [62]. In another study, miR-432 was reported to be associated with formation of curly hair of Chinese tan sheep [63]. It was also shown that miR-27a regulates the cell cycle by inhibiting the TGF-β/smad pathway [64, 65]. The related circRNAs identified in our study might play an important regulatory role in the growth and development of wool follicles in AFWS. We intend to verify this further in future experiments. Our study detected a large number of circRNAs in the skin of AFWS. These results provide a solid theoretical foundation for investigating the association between circRNAs and sheep (secondary) wool follicle development. Furthermore, candidate circRNAs chosen for our future wool follicle regulation research include circ_0005720, circ_0001754, circ_0008036, circ_0004032, circ_0005174, circ_0005519, and circ_0007826.

## Conclusion

Our study is the first to elucidate changes in wool follicles in sheep’s fetal development. RNA-Seq analysis identified 918 differentially-expressed circRNAs. Using miRanda to predict relationships between circRNAs and miRNAs, we identified 17 pairs of circRNA-miRNA. Of the identified miRNAs, miR-370-3p, miR-432, and miR-27a were reported to be associated with hair growth. The KEGG analysis of the differentially expressed genes identified six significantly enriched pathways. These include of 55 source genes. Seven of these genes, corresponding to 35 circRNAs, are involved in regulation of wool follicle growth. Seven of these 35 circRNAs were found to be differentially-expressed. These findings might provide clues that would assist future research on the molecular mechanisms of wool growth.

## Methods

### Sample preparation

The AFWS used in this study were raised in the AFWS Stud Farm of Inner Mongolia Autonomous Region and fed according to the farm’s feeding plan. Twelve healthy AFWS ewes of similar age (3–5 years old), body weight (55–60 kg), and body size were selected. Estrus of the 12 ewes was synchronized, and artificial insemination was performed during September. The ewes and lambs were anesthetized with sodium pentobarbital at a dose of 25 mg/kg by intravenous injection. After samples collection, the ewes and born lambs were released, whereas the fetuses from E90d and E120d were placed, still under anesthesia, inside a closed chamber, which was filled with 20% carbon dioxide per minute. When gas concentration had reached 80%, the fetuses died. The anesthesia procedure was performed following published protocols [66, 67].

The 2-cm-diameter skin tissue samples (about 0.5–1.0 g per fetus/lamb) were collected from the shoulder area at the three developmental stages (E90d, E120d, and Birth), three individuals for each stage, nine in total. The collected samples were placed into clean RNAase-free Eppendorf tubes and stored under liquid nitrogen, pending total RNA extraction. Skin samples were also fixed in 4% formaldehyde, and paraffin sections were prepared and stained with H&E for histological observations.

### RNA isolation and quality assessment

To extract total RNA from the nine samples, TRIzol reagent (Life Technologies, CA, USA) was used. RNase-free DNase (Tiangen, Beijing, China) was used to remove DNA contamination from the extracted RNA. RNA degradation and contamination were monitored by 1% agarose gel electrophoresis and RNA purity was measured at an OD260/280, using a NanoDrop ND-2000 instrument (Thermo Fisher Scientific, MA, USA). We also assessed RNA integrity by testing the RIN of the samples.

### CircRNA sequencing

High-throughput whole transcriptome sequencing and subsequent bioinformatics analyses were performed by Annoroad Technologies (Beijing, China) as follows: A total of 3 μg RNA per sample were used for circRNA sample preparation. The Ribo-Zero™ Gold Kit was used to remove rRNA from the samples, and different index tags were selected to build the library according to the specifications of NEB Next Ultra Directional RNA Library Prep Kit for Illumina (NEB, Ispawich, USA). The specific steps of library construction were as follows: Ribosomal RNA was removed using a kit, RNase R was added to remove linear RNA. Fragmentation Buffer was added to the reaction system to fragment the RNA, and then this fragmented RNA was used as a template for first strand cDNA synthesis, using random primers (Random Hexamers). Second strand cDNA was synthesized by adding buffer, dNTPs, RNase H, and DNA Polymerase I. After purification by QiaQuick PCR kit and elution with EB buffer, the following steps were performed: repair ending, adenine addition, sequencing linker addition, and target size fragments’ recovery (approximately 350 bp) by agarose gel electrophoresis. Uracil N-glycosylase (UNG) was then added to digest the DNA strand prior to PCR amplification. Finally, agarose gel electrophoresis was used to recover the DNA fragments of the target size. The constructed library was sequenced using Illumina X Ten and PE150 sequencing strategy.

### Sequencing analysis of circRNA

Sheep genome oar_v4.0 was selected as reference genome for comparison with the RNA-Seq data. Reads were mapped to the reference genome using the BWA-MEM method, which is fast and efficient in aligning reads, and allows mapping fragment reads to genomes as well. The raw reads generated by Illumina sequencing were processed to create clean reads by several processes, including de-junction contamination and removal of rRNA. For mapping, the BWA-MEM algorithm was first used for sequence splitting and alignment. The resulting Sam files were scanned in search of PCC (paid Chinese clipping) and PEM (paid end mapping) sites, as well as GT-AG splicing signals. Finally, sequences with junction sites were re-aligned with dynamic programming algorithm to ensure the reliability of circRNA identification. CIRI [68], an efficient and rapid tool for circRNA recognition, was also used. All subsequent analyses were based on the clean reads. The process of analyzing the circRNAs sequencing information in this study was divided into seven parts: (1) sequencing data quality control, (2) data alignment analysis, (3) circRNAs identification and classification, (4) circRNAs characteristics analysis, (5) circRNAs differential analysis, (6) differentially-expressed circRNAs source genes functions, and (7) miRNA molecular sponge analysis.

### Identification of differentially-expressed circRNAs

We used SRPBM as a normalization method to quantify the expression of circRNA. The DEseq2 [69] software was used to analyze the differentially-expressed circRNAs. The three fetuses/lambs at each stage were used as biological replicates. Differentially-expressed circRNAs were detected by comparing one stage with another. CircRNAs with *P* < 0.05 and absolute fold-change values of > 1.5 in any of the pairwise comparisons were considered to be significantly differentially-expressed. Upregulated and downregulated circRNA numbers were eventually obtained. The calculation formula of SRPBM is: $\mathrm{SRPBM}=\frac{\mathrm{SR}\ast {10}^9}{\mathrm{N}}$, where SR is the number of spliced reads, and N is the total number of mapped reads in the sample.

### Gene ontology and Kyoto encyclopedia of genes and genomes pathway enrichment analyses

Gene Ontology and KEGG pathway analyses were used to annotate the source genes of differentially-expressed circRNAs. The Blast2GO method [70] was used for GO functional analysis, while KOBAS software was used to test the statistical enrichment of differential gene expression in the KEGG pathway analysis [71]. Enrichment was considered significant in the GO term and KEGG pathway analyses when *P* < 0.05.

### Prediction of miRNAs targeted by circRNA

To explore the functions of circRNAs, predict the targeting relationship, and thus predict which of the circRNAs function as miRNAs sponges, we used miRanda V.3.3a (http://www.microrna.org/microrna/home.do) [72]. In view of known reports and extractability of the sequences, we selected only CLASSIC and ANTISENSE circRNA types for the prediction of the miRNA targeting relationship.

### Experimental validation of circRNAs

Quantitative real-time PCR (qRT-PCR) was used to validate circRNAs expression. We randomly selected seven circRNAs for validation. The expression levels of the selected circRNAs were normalized against the expression of a housekeeping gene, *GAPDH*. Primers were designed and synthesized by Sangon Biotech Co., Ltd. (Shanghai, China). Total RNA was converted into cDNA using random hexamers with Transcriptor First Strand cDNA Synthesis Kit (Roche, Australia). The qRT-PCR analysis was carried out in triplicate with iTaq™Universal SYBR@ Green Supermix (Bio-Rad, CA, USA) on a Bio-Rad CFX96 instrument (Bio-Rad, CA, USA). The total 20 μL reaction mixture contained 10 μL 2 × iTaq™Universal SYBR^@^ Green Supermix, 1 μL cDNA, 8 μL ddH_2_O, and 0.5 μL each of forward and reverse primers. The following program was used: 95 °C for 10 min; 45 cycles of 95 °C for 10 s, 60 °C for 10 s, and 72 °C for 10 s; 72 °C for 6 min. The 2^-ΔΔCt^ method was used to analyze the relative expression levels of the selected circRNAs.

To determine the resistance of the selected seven circRNAs to RNase R digestion, total RNA and RNase R (Geneseed Biotech, Guangzhou, China) were mixed together. The mix was incubated at 37 °C for 15 min, cDNA was then synthesized, and expression level of circRNAs was finally detected by qRT-PCR.

## Supplementary information

**Additional file 1 **: **Table S1. (a).** Quality report on the nine RNA samples for RNA sequencing. **(b).** The overall assessment of sequencing data.

**Additional file 2 **: **Table S2.** Total circRNAs detected at the E90d, E120d, and Birth stages.

**Additional file 3 **: **Table S3**. Differentially expressed circRNAs in the three comparison groups. (**Table S3A**: Birth compared to E90d; **Table S3B**: Birth compared to E120d; **Table S3C**: E120d compared to E90d).

**Additional file 4 **: **Table S4**. Detailed results of GO analysis for circRNAs source genes. (**Table S4A**: Birth compared to E90d; **Table S4B**: Birth compared to E120d; **Table S4C**: E120d compared to E90d).

**Additional file 5 **: **Table S5.** Detailed results of KEGG pathway analysis for source genes of identified circRNAs. (**Table S5A**: Birth compared to E90d; **Table S5B**: Birth compared to E120d; **Table S5C**: E120d compared to E90d).

**Additional file 6 **: **Table S6A.** A total of 55 source genes and their corresponding 255 circRNAs were enriched in the significantly differentially-expressed KEGG pathways. **Table S6B.** Seven source genes and their corresponding 35 circRNAs.

**Additional file 7 **: **Table S7.** Primer sequences for qRT-PCR of the seven randomly selected circRNAs.

## Data Availability

Additional data can be found in supplementary files. The RNA-Seq data was submitted to the SRA database under accession number PRJNA595784.

## Abbreviations

“H&E staining”: Hematoxylin and eosin staining
AFWS: Aohan fine wool sheep
DEG: Differentially-expressed gene
E90d, E120d, Birth: Embryonic day 90, embryonic day 120, and lamb on the day of birth
GO: Gene Ontology
KEGG: Kyoto Encyclopedia of Genes and Genomes
RIN: RNA Integrity Number
SRPBM: Spliced Reads Per Billion Mapping
