# Supplementary material for: Identification and characterization of circRNAs in the skin during wool follicle development in Aohan fine wool sheep
Source: BMC Genomics. 2020 Feb 28;21:187. doi: 10.1186/s12864-020-6599-8 (PMC7048093; doi:10.1186/s12864-020-6599-8)
Supplement: Supplementary file 1 — Additional file 1 : Table S1. (a). Quality report on the nine RNA samples for RNA sequencing. (b). The overall assessment of sequencing data. [file 12864_2020_6599_MOESM1_ESM.docx]

| **Sample** | **Total RNA(μg)** | **OD260/OD280** | **OD260/OD230** | **28S/18S** | **RIN value** |
| --- | --- | --- | --- | --- | --- |
| **E90d_1** | 34.9 | 2.0 | 1.8 | 0.9 | 7.0 |
| **E90d_2** | 60.0 | 2.0 | 1.4 | 0.9 | 7.3 |
| **E90d_3** | 45.9 | 2.0 | 1.3 | 1.2 | 8.7 |
| **E120d_1** | 24.1 | 2.0 | 1.9 | 0.5 | 6.9 |
| **E120d_2** | 64.1 | 2.0 | 1.7 | 1.1 | 7.4 |
| **E120d_3** | 23.3 | 2.0 | 1.7 | 0.6 | 7.0 |
| **Birth_1** | 49.1 | 2.0 | 1.7 | 1.0 | 7.8 |
| **Birth_2** | 40.5 | 2.0 | 1.7 | 0.8 | 8.1 |
| **Birth_3** | 37.6 | 2.0 | 1.6 | 0.9 | 7.7 |

**Table S1(a)**

**Quality report of nine RNA samples for RNA sequencing**

Note: 1, 2, 3 represent three repeated samples of each period.

**Table S1(b)**

**The overall assessment of sequencing data**

| **Sample** | **Raw Reads Number** | **Raw Bases Number** | **Clean Reads Number** | **Clean Reads Rate (%)** | **Clean Bases Number** |
| --- | --- | --- | --- | --- | --- |
| **E90d_1** | 97,989,816 | 14,698,472,400 | 89,119,760 | 90.95 | 13,367,964,000 |
| **E90d_2** | 101,765,678 | 15,264,851,700 | 96,814,604 | 95.13 | 14,522,190,600 |
| **E90d_3** | 105,656,172 | 15,848,425,800 | 100,222,114 | 94.86 | 15,033,317,100 |
| **E120d_1** | 107,338,688 | 16,100,803,200 | 101,236,646 | 94.32 | 15,185,496,900 |
| **E120d_2** | 92,789,664 | 13,918,449,600 | 88,342,554 | 95.21 | 13,251,383,100 |
| **E120d_3** | 105,558,238 | 15,833,735,700 | 95,134,062 | 90.12 | 14,270,109,300 |
| **Birth_1** | 98,448,206 | 14,767,230,900 | 88,602,182 | 90 | 13,290,327,300 |
| **Birth_2** | 90,867,370 | 13,630,105,500 | 82,438,564 | 90.72 | 12,365,784,600 |
| **Birth_3** | 95,386,326 | 14,307,948,900 | 90,373,018 | 94.74 | 13,555,952,700 |

Note: 1, 2, 3 represent three repeated samples of each period.
